# The third booster vaccination dose against COVID-19: indication for circulating SARS-CoV-2 variants

**DOI:** 10.2217/fvl-2021-0240

**Published:** 2021-11-04

**Authors:** Farid Rahimi, Amin Talebi Bezmin Abadi

**Affiliations:** ^1^Research School of Biology, The Australian National University, Canberra, Australia; ^2^Department of Bacteriology, Faculty of Medical Sciences, Tarbiat Modares University, Tehran, Iran

**Keywords:** booster immunization, COVID-19 vaccines, pandemic, SARS-CoV-2 variants

## The COVID-19 pandemic

One and half years after its discovery in Wuhan, China, COVID-19 has turned into a global public-health catastrophe [1–3]. On 13 September 2021, unfortunately 225,707,678 confirmed cases and >4.6 million deaths were reported globally [4]. To date, no hypothetical drugs have been proven to defeat SARS-CoV-2, despite some contradictory reports [5]. Thus, targeted interventions should be optimized and continued. The epidemiological countermeasures that have been variedly implemented worldwide generally include frequent handwashing, physical distancing, wearing face masks, contact tracing and banning of mass gatherings. If these were abandoned, the daily increases in COVID-19 case numbers will undoubtedly cause high hospitalization and high fatality rates [6,7]. Expectedly, complying with and upholding all the countermeasures by both people and health authorities are difficult for many reasons that have been discussed by some authors previously [8–10]. Therefore, global vaccination is the most important and effective countermeasure.

Indeed, months after the declaration of the pandemic by WHO, scientists and clinicians started employing several approaches for producing effective or prophylactic vaccines against COVID-19 [11–14]. Many vaccines have so far been produced and administered. However, equitable rollout of a rapid vaccination campaign requires implementation and assessment of procedures pertaining to mass production, storage, transport logistics, prioritization of target populations, indications of vaccine types, vaccine dosage and intervals between multiple doses [13,15,16].

### Vaccination challenges

Unfortunately, the number of identified, circulating, SARS-CoV-2 variants is alarming in a relatively large number of countries that struggle with the pandemic (supportive data representing December 2019–September 2021 can be accessed through https://nextstrain.org/ncov/gisaid/global). Timely tracking of the new variants requires sophisticated sequencing instruments and facilities. Wealthy countries have implemented routine sequencing measures to track the viral variants; however, many poor countries lack such expensive facilities to document the circulating viral variants by sequencing.

High antibody titers induced by any immunization or previous infection expectedly wane gradually – initially rapidly in the first two years following immunization or infection (reviewed in [17]). Antibody levels following BNT162b2 (Pfizer–BioNTech) and ChAdOx1 nCoV-19 (Oxford–AstraZeneca) vaccines reportedly decrease months after the double-dose vaccination [18]. Some new SARS-CoV-2 variants of interest or concern (variants of interest [VOIs] and variants of concern [VOCs]) reportedly confer substantial or complete resistance against vaccines [19,20]. A Dutch group raised alarm in a preprint about a reduction of binding and neutralizing potency of antibodies against three SARS-CoV-2 VOCs (B.1.1.7, B.1.351 and P.1) in convalescent or immunized sera from a cohort including COVID-19 patients and recipients of the Pfizer–BioNTech vaccine [19]. How long immunized subjects could present an acceptable level of antibodies or mount an effective cellular immune response against SARS-CoV-2 or its variants is uncertain. In addition, viral antigenic variations may help the virus to evade the immune response or the antibody pools that were effective against the original lineage. While mass production and distribution of any prophylactic vaccine is crucial, restoring vaccine effectiveness against a new variant may be more difficult and time demanding, considering the high possibility of frequent mutations occurring in the unstable and evolving viral genome. Information about the SARS-CoV-2 variants is scarce now; however, accepting that they may continue to circulate and predominate the pandemic is logical [21].

Briefly, two important factors challenge the success of the vaccination campaigns against COVID-19. First, the surging new viral variants have rapidly disseminated globally and may evade immune responses in already vaccinated or previously infected subjects. Second, the antibody titers wane months or years after immunization or following the first infection. These two challenges are interdependent. The future course of the pandemic will be determined by a balance between emergence of the new variants and production of the vaccines.

### Indication for a booster third-dose vaccination

May 2021 saw growing debates about a booster third vaccine dose against the virus. Presently, no clinical evidence exists to support the third dose in fully vaccinated or previously infected subjects. The rationale behind the prescription of the triple-dosing is supported mainly by the emergence of the various SARS-CoV-2 variants that have started to dominate the pandemic. Rapid emergence and dissemination of the Delta variant with high infectivity and transmissibility has prompted the experts and decision-makers to consider the third dose [22]. Some reports suggest that the third dose may confer more-effective immunity against the new SARS-CoV-2 variants [23]. The inevitable emergence of SARS-CoV-2 variants (either VOCs or VOIs) and waning levels of neutralizing antibodies in vaccinated or previously infected subjects justify the third dose months after initial and second dosing [24]. Importantly, SARS-CoV-2 and its VOCs or VOIs may continue evolving and emerging years after the 2020 pandemic. Therefore, a third dose of vaccination will better protect the vulnerable populations.

Uncertainties abound about identifying the target groups for triple-dose vaccination. On 12 August 2021, US FDA announced in a news release that “… this small, vulnerable group (immunocompromised individuals) may benefit from a third dose of the Pfizer–BioNTech or Moderna vaccines" [25]. Thus, the third dose at least for vulnerable subjects, including elderly people, immunocompromised patients and healthcare workers (who may be frequently exposed to high viral loads), may be prudent. Indications for other population groups (e.g., indigenous peoples or those with disabilities) will need to be determined, too. However, mass administration of the triple-dose vaccination must be carefully balanced with vaccination of nonimmunized populations, including those younger than 12 years of age so that double-dosing must take precedence globally. So far, Israel, Germany, USA, China, Russia and the UAE have started triple-dose vaccinations; thus, their findings on potentially boosted immunity will clarify the clinical importance of triple-dosing of SARS-CoV-2 vaccines. Indeed, the importance of triple-dosing for the young will also need to be investigated. Determining the interval between the second and third dose also will require clinical trials in different populations. Oxford–AstraZeneca, Pfizer–BioNTech and Moderna have already initiated clinical trials to examine the validity of the third booster vaccination by using different combinations. The findings of such studies are important for considering the third dose of these and other vaccines distributed globally.

## Future perspective

The global number of cases infected with SARS-CoV-2 surpassed 225 million on 13 September 2021. Cumulative epidemiological and public-health evidence along with societal, psychological and economic hardship and turmoil dictate that mass vaccination is the best countermeasure to defeat the COVID-19 pandemic. However, antibody levels are thought to wane in immunized subjects, and the durability of effective immune responses is uncertain and undoubtedly varied among individuals. Variability of individual responses to COVID-19 has been known since the beginning of the pandemic, and such a variability may be coordinated by already complex individual immune responses. Some infected patients show aggravating symptoms requiring hospitalizations, whereas some present mild or no symptoms. Meanwhile, the rationale for a third booster vaccination is supported by the urgency to defeat the circulating SARS-CoV-2 variants that have started to dominate the COVID-19 statistics worldwide. We understand that circulating SARS-CoV-2 variants will increase steadily; thus, triple-dose vaccination, at least for immunocompromised and elderly patients, is advisable. However, the effectiveness or safety of administering a third booster vaccination in fully vaccinated subjects has not been confirmed. We do acknowledge that evidence-based countermeasures are necessary to defeat the emerging viral variants, especially VOCs, and timely responses are warranted to efficiently control the pandemic curve in the coming months. Nevertheless, we tend to postulate that the third dosing would be safe in subjects who have tolerated the previous two doses. Importantly, timing of a booster mass vaccination before emergence or dominance of any new VOC could be crucial in controlling the pandemic. Considering that the SARS-CoV-2 variants will inevitably continue emerging and circulating, administering the third vaccination dose at least to the elderly and subjects with immunodeficiencies or disabilities seems sensible. Documenting the vaccine type, its booster dosing, target populations, optimal dosing intervals and the vaccination timeline is crucial for determining the vaccine effectiveness against the existing or new SARS-CoV-2 variants. Any change in the future statistics of COVID-19 may help us better understand the virus and its potential seasonally emerging variants akin to the seasonal flu or measles.
